# Profiling of Silk Sericin from Cocoons of Three Southern African Wild Silk Moths with a Focus on Their Antimicrobial and Antioxidant Properties

**DOI:** 10.3390/ma13245706

**Published:** 2020-12-14

**Authors:** Kanono Comet Manesa, Temesgen Girma Kebede, Simiso Dube, Mathew Muzi Nindi

**Affiliations:** Department of Chemistry, Florida Science Campus, University of South Africa, Roodepoort 1709, South Africa; maneskc@unisa.ac.za (K.C.M.); tgkkebede@gmail.com (T.G.K.); nindimm@unisa.ac.za (M.M.N.)

**Keywords:** sericin, amino acids, XRD, FTIR, antibacterial, antioxidant

## Abstract

Silk sericin was extracted from the cocoons of three Southern African wild silk moth species, namely *Gonometapostica*, *G. rufobrunnae* (Lepidoptera: Lasiocampidae), and *Argema mimosae* (Lepidoptera: Saturniidae); these three sericin extracts were analysed to determine the relationship that exists between their chemical structures and their functional properties. The relationship was investigated by utilising several methods that include the determination of the amino acid composition, and characterisation of the secondary structures with Fourier transformation infrared spectroscopy (FTIR) and X-ray diffraction (XRD). The antibacterial properties of these three sericin extracts were evaluated by an agar well diffusion assay with three Gram-positive bacteria (*Bacillus subtilis, Staphylococcus aureus*, and *Staphylococcus epidermidis*) as test microorganisms; and, lastly, the antioxidant properties of the three sericin extracts were determined using several scavenging methods that include the 2,2-diphenyl-1-picrylhydrazyl radical (DPPH), 2,2′-azinobis-(3-ethylbenzothiazoline-6-sulfonic acid) (ABTS˙^+^), and the ferric reducing antioxidant power (FRAP) assay. The amino acid composition in the silk sericin extracts from *G. postica*, *G. rufobrunnea*, and *Argema mimosa* in terms of the polar/non-polar ratio (P/NP) was found to be 65:35, 56:44, and 59:41, respectively. The FTIR spectra of these three silk sericin extracts showed distinct major bands such as amide A (3265 cm^−1^), amide B (3062 cm^−1^), amide I (1644 cm^−1^), amide II (1538 cm^−1^), and amide III (1244 cm^−1^). The XRD patterns of the silk sericin extracts revealed both amorphous and α-helical structures, with small crystalline regions. All three silk sericin extracts presented potent antibacterial efficacy against the three Gram-positive bacteria and were found to have excellent antioxidant activities against the tested free radicals.

## 1. Introduction

Silk protein is a natural fibre produced by the silkworm to form a cocoon for protection during the pupal stage against various threats. Silk fibre consists of two distinct proteins, namely the fibroin and sericin. Silk sericin is a glue-like protein, which binds the fibroin fibres into an intact cocoon. Sericin protein amounts to 20–30% of the total weight of the silkworm cocoon while fibroin protein makes up the rest [1]. Silk sericin protein is considered as a waste product in the silk industry, and a large amount is usually discarded. However, it has been found to possess important biochemical properties [2]. It has 18 amino acids that mainly contain polar side-chains made of hydroxyl, carboxyl, and amino groups. These functional groups allow sericin protein to attain easy cross-linking, blending, and copolymerisation with other natural and synthetic polymers to produce biomaterials with enhanced properties. The high content of polar amino acids, namely serine, aspartic acid, and glutamic acid contribute towards the hydrophilic nature of sericin protein [3]. It is assumed that polar amino acids also contribute towards the essential properties of silk sericin, such as antioxidant and antibacterial properties, anti-tyrosinase activity, ultraviolet (UV-B) rays resistance, and regulating moisture and solubility in hot water [4,5,6]. For example, the antioxidant action of silk sericin is shown by its ability to suppress lipid peroxidation, to inhibit tyrosinase activity in-vitro, to inhibit apoptosis induced by UV-B, and by chelating trace elements such as Cu, Fe, and Zn [7,8].

These aforementioned activities were attributed to the high amount of polar amino acids with hydroxyl groups, mainly serine and threonine, which are known to be abundant within the secondary structure of the silk sericin. Therefore, the strength of silk sericin antioxidant properties is primarily due to its amino acid composition that allows for the scavenging of free radicals [9,10]. In addition, the silk sericin layer contains other substances such as the organic pigments which are known for their biological properties like antioxidant and anti-tyrosinase activity. For instance, coloured cocoons exhibit additional higher anti-tyrosinase activity to white-shelled cocoons. This is as a result of organic pigments accumulating within the sericin layers of coloured silk cocoons [11]. Therefore, polar amino acids and the pigments are both responsible for antioxidant properties. The unique physicochemical properties of silk sericin are affected by the method of removing sericin from the silk fibroin (degumming), thus influencing its antioxidant activity.

Sericin is reported to possess antimicrobial activity against a few microorganisms. For example, silk sericin-coated fabric was found to have a high degree of bactericidal activity against test organisms such as *Escherichia coli* and *Staphylococcus aureus* [12]. In another study, silk sericin demonstrated antimicrobial efficiency for polluted air filters that were covered with sericin prior to use [5]. However, the aforementioned studies give no clarity about the mode of action used by sericin against the test microorganisms [13,14,15]. The aim of this study was to gain an understanding of the relationship between the functional structure of sericin and its aforementioned biological properties. This will provide merits of the three wild sericin when compared to domesticated and other natural biopolymers, thus allowing for the effective utilisation of the material in different biomedical fields.

## 2. Materials and Methods

Twenty L-amino acid standards, Dabsyl-Cl, and Whatman syringe filters polyvinylidene fluoride (PVDF) membrane (pore size 0.45 μm) used in this study were purchased from Sigma-Aldrich (Steinheim, Germany).Hydrochloric acid, sodium carbonate, sodium hydrogen carbonate, sodium acetate, sodium hydroxide pellets were all purchased from Merck (Darmstadt, Germany), while 2,2-diphenyl-1-picrylhydrazyl radical (DPPH), 2,2′-azinobis-(3-ethylbenzothiazoline-6-sulfonic acid) (ABTS), Folin–Ciocalteu reagent, 6-hydroxy-2,5,7,8-tetramethylchroman-2-carboxylic acid (Trolox), gallic acid (GA), and FeCl_3_ were obtained from Sigma-Aldrich (Steinheim, Germany). All the chemicals and reagents were of analytical or high-performance liquid chromatography (HPLC) grade (95–99.9% purity). A diode-array detector (DAD) was used. The ultra-high purity (UHP) water used for preparation of solutions was generated from a Milli-Q system with resistivity of 18.2 MΩcm (Millipore, Billerica, MA, USA). The Mueller–Hinton agar used to prepare the agar plates was purchased from Merck (Eppelheim, Germany).

### 2.1. Silk Sericin Samples

The sericin derived from three wild Southern African silkworm cocoons was originally degummed. The *Gonometa postica* cocoons were harvested in the area near Ganyesa, a rural village located in North West Province, South Africa. The *Gonometa rufobrunnae* cocoons were obtained near Shashe-Mooke, a rural village located in the Central District of Botswana. The *Argema mimosa* cocoons were collected from the Manzini region of Swaziland.

### 2.2. Amino Acid Analysis

Amino acids were obtained by hydrolysing 10 mg of silk sericin samples in 2 mL of 6 M HCl at 110 °C for 24 h and, thereafter, 50 µL of the resulting sericin hydrolysates were transferred into micro-centrifuge tubes and then derivatised with Dabsyl-Cl. Afterwards, the derivatised silk sericin hydrolysates were dried and re-dissolved in 4 mL of ethanol. The amino acid analysis was carried out using an Agilent 1200 HPLC-DAD system (Agilent Technologies, Waldbronn, Germany). ChemStation software (version 4.3.) was used to control the instrument and process the data. An Agilent ZORBAX Eclipse XDB-C18 (4.5 × 150 mm, 5 µM) column was used for chromatographic separation.

### 2.3. Fourier Transform Infrared (FTIR) Analysis of Silk Sericin Proteins

An attenuated total reflection-Fourier transforms infrared (ATR-FTIR) spectrophotometer (Bruker Corporation, Ettlingen, Germany) with a single-reflection diamond attachment was used to determine the secondary structural transition of three pure sericin powdered extracts. All spectra were obtained at room temperature, within a wave-number range of 600–4000 cm^−1^ with 32 scans at 4 cm^−1^ resolution.

### 2.4. X-ray Diffraction (XRD) Analysis of Silk Sericin Proteins

The crystallinity of pure sericin powdered samples was analysed with a Rigaku Smart Lab 9 kW, high-resolution X-ray diffraction system (Rigaku, Neu-Isenburg, Germany) using CuKα radiation for the determination of diffraction intensity curves. These were obtained at a *λ* = 1.5 Å for 2θ from 10° to 60° at a scanning rate of 0.0015° s^−1^. Voltage and current of the X-ray source used were 45 kV and 200 mA, respectively.

### 2.5. Silk Sericin Antioxidant Activity

#### 2.5.1. Total Phenolic Content of Silk Sericin Proteins

The total phenolic content of sericin was determined according to a method of Slinkard and Singleton [16] which involves the use of Folin–Ciocalteu reagent and gallic acid as standard. A 0.1 mL aliquot containing 1 mg of sericin was transferred to a 50 mL volumetric flask, and 1 mL of Folin–Ciocalteu reagent was also added. The flasks were shaken carefully, and after 3 min, 3 mL of (2%) Na_2_CO_3_ was added, and the mixture made up to volume with ultra high pure (UHP) water. Subsequently, the mixtures were shaken on a shaker for twohours at room temperature, and the absorbance was measured at 760 nm at 25 °C using a spectrophotometer (Perkin Elmer Lambda 35 ES UV/Vis (ultraviolet–visible; Boston, MA, USA)). The same method was repeatedfor all gallic acid standard solutions (0–100 mg/L). The tests were all performed in quintuplet, and the obtained results were averaged. The total phenolic content in silk sericin was expressed as mg GAE/100 g of sericin extracted from each of the three silk moths trains.

#### 2.5.2. DPPH (2,2-Diphenyl-1-Picrylhydrazyl) Radical Scavenging Activity of Silk Sericin Proteins

The original Blois [17] DPPH method with modifications was used to determine the activity of sericin as a free radical scavenger. A 0.1 mM solution of DPPH in methanol was prepared, and 1 mL of this solution was added to 3 mL of different concentrations of sericin (15, 30, 45, 60, 75, 90, and 120 μg) and the reference material (Trolox). The mixtures were vigorously shaken using a vortex and then allowed to stand at ambient temperature for 30 min. The absorbance was measured at 517 nm. These tests were carried out in triplicate, and the results averaged. The percentage inhibition was calculated by comparing the absorbance values of the control and sample. The radical-scavenging activity (*S*) at different sericin concentrations was calculated by Equation (1):

(1) S=100×(1−Ax/Ao)

where *Ax* is the absorbance of the solution in the presence of sericin, and *Ao* is the absorbance of the DPPH solution in the absence of the sericin.

### 2.6. Reducing Power of Silk Sericin

The reducing power of the three silk sericin samples was established using the ferric-reducing antioxidant power (FRAP) assay of Oyaizu [18] with minor modifications. Silk sericin solutions with different concentrations (1.0, 2.0, 4.0, 6.0, 8.0, and 10 mg/mL) were prepared in UHP water. For each solution, a 1.0 mL aliquot was mixed with 2.5 mL of 0.2 M phosphate buffer (pH 6.7) and 2.5 mL of 1% potassium ferricyanide. The mixtures were gently swirled and incubated for 20 min at 50 °C. This was followed by the addition of 2.5 mL of 10% trichloroacetic acid (TCA) to each mixture. The mixtures were centrifuged at 5000 rpm for 10 min, and then 2.5 mL of the supernatant were transferred into test tubes that contained 2.5 mL of UHP water and 0.5 mL of 0.1% ferric chloride (FeCl_3_·6H_2_O). The mixtures were homogenisedwith a vortex, and then the absorbance was measured at 700 nm against the reagent blank. Trolox was used as a positive comparative control.

### 2.7. 2,2′-Azinobis-(3-Ethylbenzothiazoline-6-Sulfonic Acid) (ABTS˙^+^) Radical Cation Assay

The ABTS˙^+^ radical cation scavenging activity of the silk sericin extracts was determined utilisingthe method of Re et al. [19]. The ABTS˙^+^ radical cation was produced by the reaction of 2.5 mL of 7 mM ABTS˙^+^ solution and 2.5 mL of 2.4 mM potassium persulphate placed in the dark for 12 h at room temperature. The ABTS˙^+^ stock solution was diluted with a 0.2 M phosphate buffer solution (PBS) to accomplish an absorbance of 0.700 ± 0.05, and then about 1 mL of diluted ABTS˙^+^ solution was mixed with 0.5 mL of silk sericin samples. About 30 min after mixing, the absorbance was measured at 734 nm. The ABTS˙^+^ scavenging efficacy (*S*) at 734 nm was calculated using Equation (2):

(2) S (%)=100×(1−As/Ac)

where *Ac* is the initial concentration of the ABTS˙^+^ and *As* is the absorbance of the remaining concentration of ABTS˙^+^ in the presence of silk sericin.

### 2.8. Antibacterial Assays

#### 2.8.1. Preparation of Bacterial Inoculum

The following bacterial strains were used to assess the antibacterial activity of the three sericin samples: *Bacillus subtilis* (ATCC 6633), *Staphylococcus aureus* (ATCC 25923) *Staphylococcus epidermidis* (ATCC 27738), *Escherichia coli* (ATCC8739), and *Salmonella enterica* serovar Typhimurium (ATCC 14028). The bacterial strains were provided by the Department of Food Science at the Tshwane University of Technology (RSA).

#### 2.8.2. Preparation of Silk Sericin Solutions for Evaluation of Antibacterial Activity

The stock solutions of the three sericin extracts were prepared at concentrations of 5, 10, 20, 40, and 60 mg/mL with 100 µL of 2% Aliquat 336 added as a carrier solvent. The experiment was conducted at different levels of pH (3.0; 4.0; 5.0; 7.0 and 9.0) of silk sericin solution. The sericin solution mixtures were dissolved and adjusted to their designated pH’s with 0.1 M HCl, while L-ascorbic acid (vitamin C) was used as a positive control.

### 2.9. Agar Well Diffusion Assay

The agar well diffusion assay method was used to evaluate the antibacterial activity of the sericin extracts. Each bacterial culture was sub-cultured in a nutrient broth at 37 °C for 24 h to obtain pure isolates. Pure isolate inoculums containing approximately 1 × 10^8^ colony-forming units (CFU)/mL were evenly spread on Mueller–Hinton agar plates using a sterile loop to obtain a homogeneous growth, and the plates were allowed to dry for 10 min. The agar wells were prepared by carefully cutting 6 mm diameter wells from the agar plates, and then 50 μL of the sericin solutions were poured into the wells. The agar plates were then incubated at 37 °C for 24 h. The inhibition zones around the agar wells were recorded using a micrometer. Bacterial growth was determined by measuring the diameter of thezone of inhibition, including the well-size. The minimum inhibitory concentration (MIC) assay was done in triplicate and the data recorded are the average of the three independent tests.

#### Statistical Analysis

All statistical analyses were conducted in quintuplet, and the data expressed as the mean ± standard deviation (SD) for n = 5. Analysis of data was performed using variance at the 95% confidence interval, standard error, fitted regression equation, and coefficient of determination (R^2^) (ANOVA, Microsoft Excel Office 2010). The results were statistically significantwhen *p*-values were less than 0.05 (*p* < 0.05). ANOVA was also used to measure the level of antioxidant activity (at EC_50_) and antibacterial activity (inhibition zones in mm).

## 3. Results

### 3.1. Amino Acid Composition

The results of amino acid chemical composition of the sericin protein derived from *G. postica*, *G. rufobrunnea*, and *Argema mimosa* are presented in Table 1. Amino acids such as serine, aspartic acid, and glutamic acid were found to constitute 78–80% of the total polar amino acids in each of the sericin strain. The three silk sericin extracts were found to have a large amount of acidic amino acids compared to their basic counterpart. The total amount of amino acids with hydroxyl groups (Ser-OH, Thr-OH, and Tyr-OH) in *G. postica* sericin was found to constitute 39%, while it amounted to 27% and 25%, respectively, in *G. rufobrunnea* and *Argema mimosae* sericin. Silk sericin from *G. postica* was found to have a polar/non-polar amino acid ratio of 65:35, while the polar/non-polar amino acid ratios of *G. rufobrunnea* and *Argema mimosa* sericin were found to be 56:44 and 59:41, respectively. Several studies have reported comparable polar/non-polar amino acid ratios in silk sericin from different strains (*Bombyx mori*, 79:21; *Antheraea mylitta*, 75:25; *Cricula trifenestrata*, 69:31; and *G. rufobrunnea*, 59:41) [20,21,22].

Three silk sericin extracts (*B. mori*, *A. mylitta*, and *C. trifenestrata)* were found to have higher polar/non-polar amino acid ratios compared to the three silk sericin extracts investigated in this study. The *G. rufobrunnae* sericin extract analysed in this work has a polar/non-polar amino acid ratio comparable to the results reported by Freddi and co-workers [21]. In the case of amino acids such as serine, glycine, glutamic acid, and aspartic acid, both *G. postica* and *G. rufobrunnae* sericin extracts in this work were found to be comparable to or even possessing higher ratios than those previously reported in the literature.

### 3.2. FTIR Analysis

**Figure 1:**
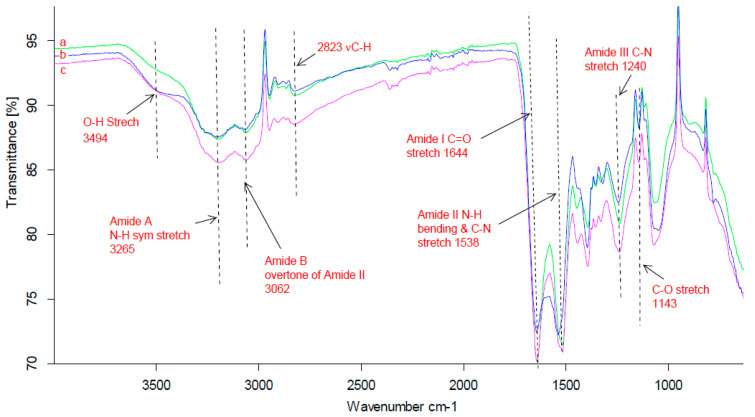
Infrared (IR) spectra of three sericin extracts obtained from cocoons of (**a**) *G. rufobrunnea* (**b**) *G. postica* and (**c**) *Argema mimosae*.

Secondary structural transitions of the three silk sericin extracts were studied using Fourier transform infrared spectroscopy. The results presented in Figure 1 exhibit characteristic absorptions of silk sericin protein including major amide absorption bands associated with its infrared spectra. Some of the apparent vibration bands associated with sericin protein amide I arise mainly from primary amides at 1643 to 1644 cm^−1^ which are ascribed to the C=O stretching vibration [23]. Amide II bands at 1538 to 1540 cm^−1^ are ascribed to N–H bending and C–N stretching vibrations, respectively [24]. All amide III bands found at around 1240 cm^−1^ are attributed to C-N stretching coupled to N–H bending vibration and usually appear as a weak band [25]. Sericin has a broad amide A band at 3265 cm^−1^ and a B band at 3062 cm^−1^ for a hydrogen bond [26]. The amide A band includes the free and bonded absorptions of O–H (stretching of serine hydroxyl groups) and N–H stretching vibration groups, while the amide B band is accredited to the first overtone of the amide II vibration originating from Fermi resonance [27]. In addition, the three silk sericin extracts were found to have a flat broad O–H (stretching of alcohols) band at 3495 cm^−1^.The three sericin extracts in this study were found to have amorphous structures due to the contribution of random coil and α-helical conformations with a minimal amount of *β*-sheets conformation, similar to previously reported results [28,29,30].

### 3.3. X-ray Diffraction (XRD) Analysis

**Figure 2:**
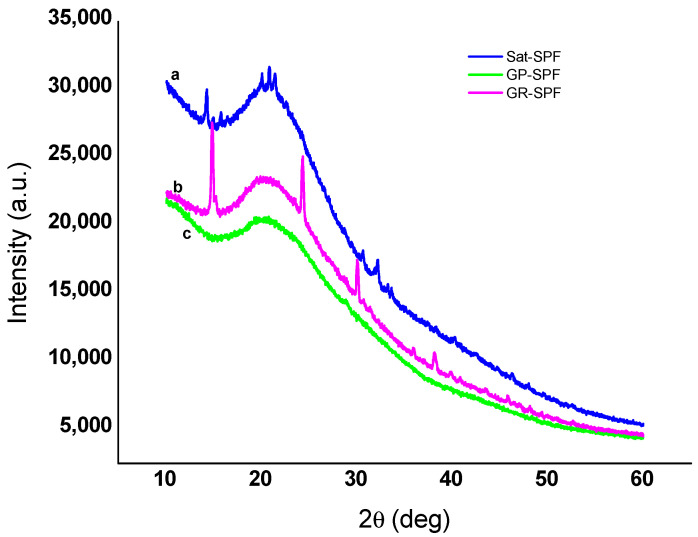
X-ray diffraction (XRD) diffractograms of sericin obtained from (**a**) *Argema mimosae*; (**b**) *G. rufobrunnea*; and (**c**) *G. postica* cocoons.

The crystalline structure of the three sericin extracts was determined by X-ray diffraction analysis. Figure 2 shows the XRD diffractograms of the three sericin extracts obtained from *Argema mimosae*, *G. rufobrunnea*, and *G. postica* cocoons, and provides an account of their comparable secondary structures. *Argema mimosae* sericin shoulder peaks were evident at around 2θ = 14.2°, 30.6, 32.1° and a major diffraction peak, which is divided into three minor peaks at 2θ = 20.0°, 20.8°, and 21.4°, while *G. rufobrunnea* sericin was found to have shoulder peaks at around 2θ = 14.8°, 24.2°, 30.0°, 38.0°, and a major peak at 2θ = 20.7°. In the case of *G. postica* sericin, a clear contrast was observed, where the shoulder peaks are not present, but only a major peak at 2θ = 20.4° was observed. The peaks obtained for sericin extracted from *Argema mimosae* and *G. rufobrunnea* strains demonstrate that these two sericin extracts contain both amorphous aggregates and α-helical structures with small crystalline regions. These findings were confirmed by the random coil and negligible amount of *β*-turn conformations obtained in the FTIR spectra ((a), (b), (c) in Figure 1) results. The broad peak of *G. postica* sericin indicates its amorphous nature (α-helix and random coil conformations), and it is due to its high polar amino acid composition. Previous studies have also reported similar results, where both random coils and a few *β*-sheets represented amorphous and crystalline regions, respectively [31,32].

### 3.4. Silk Sericin Antioxidant Properties

#### 3.4.1. The Total Phenolic Content of Silk Sericin Proteins

**Figure 3:**
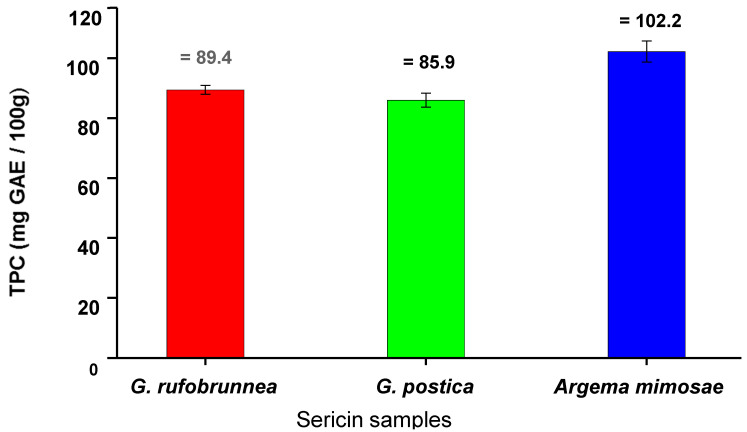
The total phenolic content expressed as mg gallic acid equivalent (GAE)/100 g of each of the three sericin extracts.

The total phenolic contents of three silk sericin samples extracted from *G. postica, G. rufobrunnea*, and *Argema mimosa* cocoons were analysed using the Folin–Ciocalteu colorimetric assay, in which results are expressed as gallic acid equivalents (GAE). The choice of gallic acid (GA) as the standard was based on its stability and purity. The stability of gallic acid standard stock solution was found to lose less of their original value over a period of two weeks when tightly closed and refrigerated at less than 2 °C. A gallic acid standard curve with a linear *regression* line equation y = 0.005x + 0.002 and coefficient of determination R^2^ = 0.999 were achieved. The linearity of the graph was further confirmed by the *p*-value that is *p* < 0.05 and thus proving that the gallic acid standard curve had a significant relationship between the variables (x and y) in the linear regression. Figure 3 shows the total phenolic content results of the three sericin extracts. *Argema mimosae* sericin was found to have the highest phenolic content (102.2 mg GAE/100 g), followed by *G. rufobrunnae* (89.4 mg GAE/100 g), and *G. postica* (85.9 mg GAE/100 g).

#### 3.4.2. DPPH Radical Scavenging Activity of Silk Sericin Proteins

**Figure 4:**
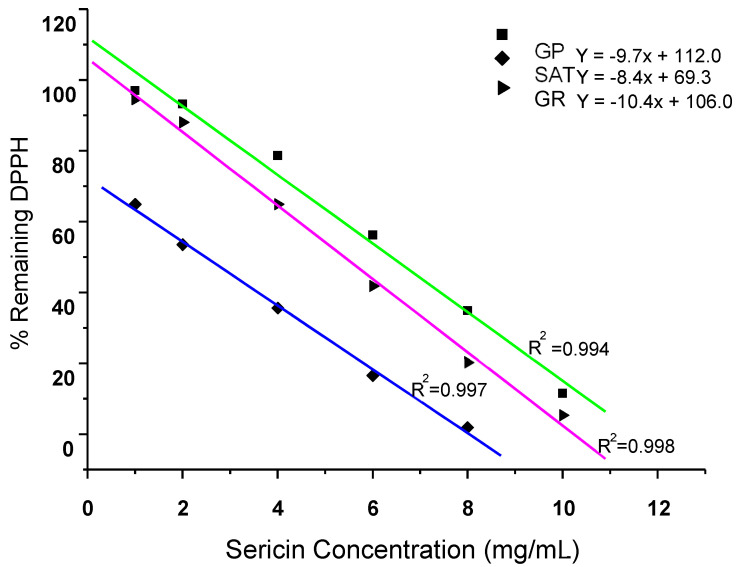
A dose-response curve fitted with a line regression sericin concentration vs.% DPPH scavenged.

Figure 4 presents the DPPH radical-scavenging ability of the three silk sericin extracts by dose-response fitted linear regression curve over a concentration range of 2.0–10 mg/mL. The mean concentrations of antioxidant necessary to decrease the initial DPPH concentration by 50% (EC_50_) were obtained from the dose-response curves. The linear regression generated coefficients of determination (R^2^) ranging from 0.994 to 0.998. The dose-response curves show that the strength of DPPH radical scavenging decreases as the concentration of silk sericin increases; this was found to be the case for sericin extracted from all three silk moth strains.

#### 3.4.3. DPPH Scavenged

Table 2 shows the EC_50_ values of the three silk sericin extracts needed to decrease the DPPH radical concentration by 50%. The EC_50_ is inversely proportional to the antioxidant capacity of the sericin concentration. This means that the lower the EC_50_ values of a particular sericin strain, the higher will be its antioxidant activity [33,34]. The EC_50_ values obtained for silk sericin derived from *G. postica, G. rufobrunnae* and *Argema mimosae* were 6.39 ± 4.1, 5.40 ± 1.5, and 2.31 ± 2.1 mg/mL, respectively. The highest EC_50_ values were found in both of the *G. postica* and *G. rufobrunnae* sericin, while a lower value was found in *Argema mimosae* sericin, which means that it has higher antioxidant activity against the DPPH radical than the sericin derived from the two *Gonometa* strains [34]. Trolox is a pure compound and is known to be a potent antioxidant [35]; it was used as a positive comparative reference standard to the three silk sericin extracts. In comparison with Trolox, *Argema mimosa* sericin displayed a comparable radical scavenging activity, when compared to the moderate scavenging activities observed in the sericin obtained from the two *Gonometa* strains. The higher DPPH radical scavenging values recorded for the *Argema mimosae* sericin are associated with its high total phenolic content and the additional effect due to the presence of carotenoid and flavonoid pigments (dark brownish colour). Both *G. postica* and *G. rufobrunnea* sericin showed slightly lower total phenolic contents (as shown in Figure 3), and pigments (yellowish colour), hence their moderate scavenging activities.

Generally, the assumed mechanism is that the silk sericin donates a hydrogen atom to the DPPH radical, thus converting it into a more stable form. This action is depicted by colour changes that took place when the original purple colour of DPPH radical is changed to a yellowish colour, signifying that the silk sericin has thoroughly scavenged the DPPH radical.

#### 3.4.4. ABTS˙^+^ Radical Scavenging Efficacy of Silk Sericin Proteins

The ABTS assay was employed to further test the relative antioxidant efficacy of the three silk sericin extracts due to its speed, sensitivity and user-friendly nature [36]. The method uses decolourisation of an organic radical cation derived from ABTS as a criterion to evaluate the interaction between silk sericin and the ABTS˙^+^ radical cation, which has a distinctive colour displaying maxima at 734 nm [37].

**Figure 5:**
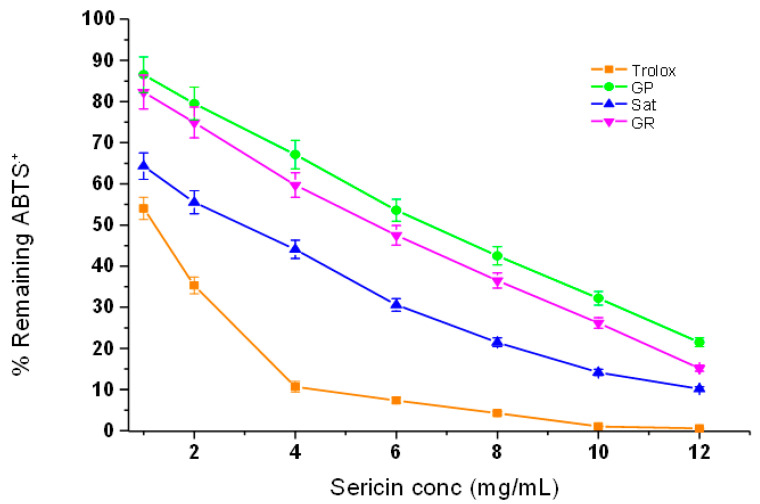
Dose-response curve of three silk sericin extracts and Trolox (control) at different concentrations vs. the ABTS˙^+^ radical.

Figure 5 presents the antioxidant effects achieved by measuring the percentage of remaining absorbance of the ABTS˙^+^ radical after being quenched by a series of silk sericin concentrations (1.0–10.0 mg/mL). Trolox was used again as a positive comparative control. As expected, the results obtained showed that Trolox, which is a pure compound, has superior antioxidant activity compared to those of the three silk sericin extracts. However, *Argema mimosae* sericin presented much better results for scavenging the radical cation than both *G. postica* and *G. rufobrunnea* sericin. The antioxidant strength of *Argema mimosa* sericin to scavenge the ABTS˙^+^ radical cation is believed to be as a result of its high amount of total phenolic content (TPC) (Figure 3) and the additional supplementary effect of carotenoid and flavonoid pigments found within the silk sericin layers.

The role played by these pigments supports the findings presented in Figure 4, which shows that the dark brownish pigments of *Argema mimosa* sericin contribute further towards its high scavenging potency compared to a yellowish pigment found in *G. postica* and *G. rufobrunnea* sericin, which offers them a moderate scavenging activity. In addition, the hydroxyl groups in the amino acids side-chains (such as cysteine, tyrosine, and histidine) that form part of the primary structure of silk sericin are also known to be responsible for reducing the blue-green ABTS˙^+^ radical cation back to its colourless neutral form via their hydrogen or electron-donating action [38,39]. Table 2 also shows the EC_50_ values of the three sericin extracts obtained for ABTS˙^+^ radical cation scavenging.

### 3.5. Reducing Power of Silk Sericin Proteins

**Figure 6:**
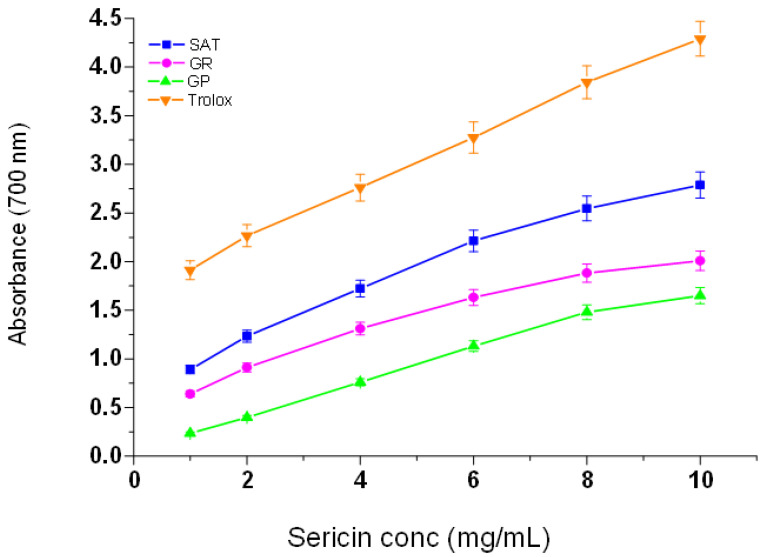
Reducing power of sericin extracted from the three wild silk moth strains and Trolox (control) at different concentrations.

The potential antioxidant activity of a test sample is indicated by its reducing power of converting Fe^3+^ to Fe^2+^ [40]. The results in Figure 6 show higher absorbance values for Trolox, which are an indication of the higher reducing power of Trolox compared to the silk sericin derived from the three wild silk moth strains. It is further observed that the reducing powers of the sericin extracted from the three wild silk moth strains increase with an increase in their concentrations. *Argema mimosae* sericin shows high reducing power compared to both *G. postica* and *G. rufobunnea* sericin. The absorbance values of Trolox are very high when compared to those obtained for the sericin extracted from the three silk moth strains, which is an indication of its greater reducing power. However, the reducing power of *Argema mimosa* sericinwas fairly high when compared with that of Trolox, and much higher than that of the sericin derived from the *Gonometa* strains (Figure 6).

The results obtained of silk sericin reducing power show a similar pattern as the cases of the DPPH and ABTS˙^+^ radical scavenging methods. The reducing property of silk sericin is associated with the presences of amino acid side-chains with abundantly found hydroxyl groups and the secondary metabolites (pigments) that exert their action of breaking the free radical chain by hydrogen- or electron-donating action. In general, the antioxidant activities of the three silk sericin extracts observed in this study are to a certain degree comparable to those previously reportedin the literature for *Samia ricini* (Eri), domesticated *B. mori*, and for sericin extracted from other wild silk moth strains [41,42].

### 3.6. Silk Sericin Antibacterial Activity

#### Agar Well Diffusion Analysis

**Figure 7:**
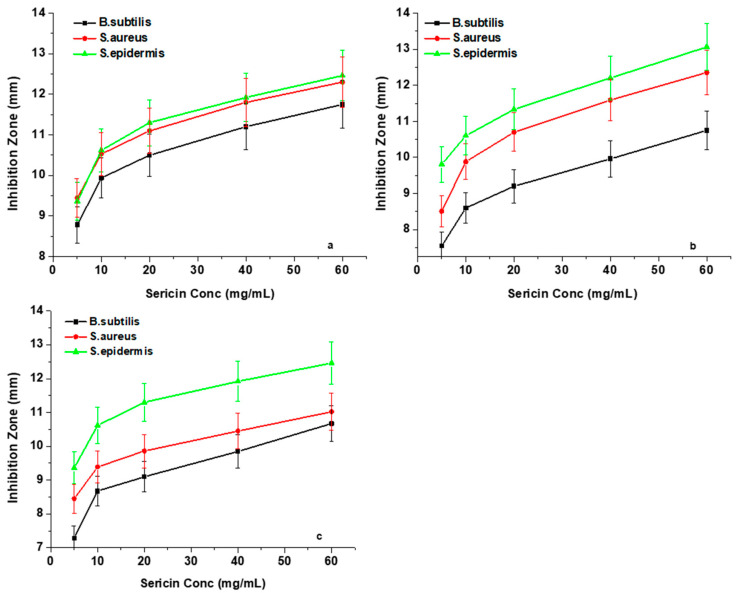
Antibacterial effects of the three sericin extracts): (**a**) *G. postica*; (**b**) *G. rufobrunnea*; and (**c**) *Argema mimosae*: Sericin concentration changes plotted against inhibition zone diameters for three microorganisms (*B. subtilis, S. aureus*, and *S. epidermidis*).

This analysis primarily focused on determining whether the two factors, namely the silk sericin concentration and the pH of sericin solution, have any influence on the antibacterial properties of the three sericin extracts. Inhibition zone plots in Figure 7a–c show the antibacterial effects that occur with silk sericin concentration changes against the three Gram-positive bacteria (*Bacillus subtilis*, *Staphylococcus aureus*, and *Staphylococcus epidermidis*). The results showed that an increase in the concentration of sericin led to a higher antibacterial efficacy against the three test Gram-positive bacteria. The inhibition activity values of all the sericin samples increased rapidly at concentrations of between 5 mg/mL and 10 mg/mL. However, beyond the concentration of 10 mg/mL, a steady increase in inhibition zone diameters was observed.

This antibacterial behaviour of silk sericin protein is explained according to the spatial arrangement of its amino acid side-chains [43]. Silk sericin protein at low concentrations provides a better molecular distribution in the solvent with a relatively small number of interactions between its neighboring chains, resulting in maximisation of the charged sites that are available for outside coupling [44]. However, in the case of higher concentrations, the inhibition zone plots present a steady increase, which represents a gradually diminishing silk sericin antibacterial potency to inhibit bacterial growth. The reason is because, at a high concentration, the development of hydrogen and covalent bonds amongst the functional groups increases, resulting in a reduced dispersion, causing the structure to have a densely overlapping coiled conformation, which subsequently establishes spatial restrictions to charged functional groups thus hindering binding to bacterial cell walls [45,46]. In this study, a minimum inhibitory concentration (MIC) of 10 mg/mL of sericin solution was found to be appropriate for bacteriostatic effects.

**Figure 8:**
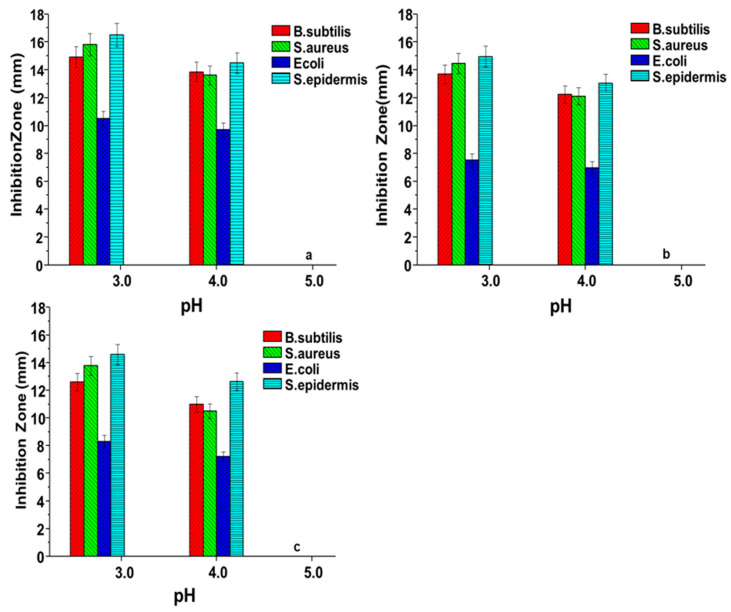
Antibacterial (inhibition zone) effects of change in the pH of the three sericin extracts (**a**) *G. postica*; (**b**) *G. rufobrunnea*; and (**c**) *Argema mimosae*: Inhibition zone diametersplotted against four microorganisms (*B. subtilis, S. aureus*, *S. epidermidis*, and *E. coli*).

Figure 8a–c show the influence of pH on the antibacterial activities of the three sericin extracts when examined against the four test microorganisms. The results did not include 2% Aliquat 336 effects as only 100 µL was added as a carrier solvent. The effects of blank samples were studied prior to application and they had no inhibition activity. The experiment was conducted at different levels of pH (3.0; 4.0; 5.0; 7.0 and 9.0) of silk sericin solutions and the reason for this was to cover a broad pH range. Sericin protein can be positive or negative based on the pH of the solution. The important point to remember is that in a pH condition below its isoelectric point, the protein will carry net positive charge and, above its isoelectric point, the protein will carry a net negative charge. The results obtained showed that acidic silk sericin protein solutions with pH 3.0 and 4.0 have inhibiting potential against the three Gram-positive bacteria. The largest inhibition zones were observed against *S. epidermidis*, followed by *S. aureus* and *B. subtilis*, respectively. The results confirm that the carboxyl functional groups of silk sericin become protonated in acidic pH, leaving sericin with positively charged amino acid side-chains, which causes the antibacterial activity of the silk sericin molecule. Therefore, the antibacterial activity of sericin could originate from its cationicamino acidside-chains, brought about by its positively charged NH_3_^+^ groups at acidic pH [47,48]. The polycationic behaviour is assumed to be the primary factor contributing to its possible mechanism of interaction with the negatively charged bacterial cell wall. This interaction affects the rigidity and stability of the peptidoglycan, producing leakage of proteinaceous and other intracellular constituents, finally resulting in bacterial growth inhibition [49,50]. The Gram-positive bacterial cell has a high internal osmotic pressure, and without a rigid cell wall, their cells burst resulting in a loss of internal cellular contents [51]. Sericin solutions with pH > 4 (at pH 5; 7; and 9) result in loss of antibacterial efficacy due to deprotonation of −NH_3_^+^ ions into a neutral NH_2_ group, which does not support interaction with the negatively charged cell wall.

The Gram-positive bacterial cell wall (thick outer peptidoglycan) easily interacts with a positively charged sericin. However, in the case of the Gram-negative (*E. coli*) bacterial cell wall, an outer layer of phospholipids renders it difficult for a positively charged silk sericin to reach the thin inner peptidoglycan layer. Hence sericin has a negligible antibacterial effect against the Gram-negative bacteria [52,53].

## 4. Conclusions

The three silk sericin extracts investigated showed comparable results with the domesticated *B. mori* sericin and other commonly used biopolymers (alginates, chitosan, collagen, fibroin, gelatine, etc.). This similarity is observed when the three silk sericin extracts were assessed regarding the relationship between the chemical structure and their functional properties. The silk sericin extracts were found to contain a large amount of polar amino acids, with hydroxyl groups contributing towards its hydrophilicity. In addition, these hydroxyl groups were found to be useful for the modification of sericin characteristics during the biological activity assay. From the FTIR spectra, the sericin extracted from the three wild silk moth strains was found to be composed of a large amount of amino acids with nucleophilic (carboxylgroup, hydroxylgroup, amino group, thiolgroup) side-chains, which are characteristics that distinguish sericin protein from other proteins. Additionally, the FTIR spectra also showed distinct major peaks (such as amide A, amide B, amide I, amide II and amide III) that are almost similar to those found in mulberry and other non-mulberry silk sericin proteins. These functional groups provide silk sericin with high chemical reactivity and are the probable basis of its biological functions, which include antioxidant and antibacterial activity. The results obtained confirm that the silk sericin extracts that presented high antioxidant activity displayed comparatively high total phenolic contents, which are almost similar to other non-mulberry silk sericin extracts. In addition, it was observed that the pigments found in the silk sericin layer of the cocoons offered supplementary antioxidant effects to each of the three silk sericin extracts.

Moreover, the three silk sericin extracts were found to have antibacterial efficacy against the three Gram-positive bacteria. The results demonstrate that the optimisation of the three silk sericin concentrations and the pH of the sericin solution influence the inhibition of the three inoculated Gram-positive bacterial strains. From the overall findings, it is clear that the sericin extracted from the three wildsilk moth strains offers potential for use in biomedical applications. Furthermore, method improvements are necessaryto mitigateits degradation as a result of harsh conditions during the process of degumming from the silk fibroin protein.

## Figures and Tables

**Table 1:** Percentage compositions (mol.% ± standard deviation (SD)) of amino acids from the three sericin extracts (*G. postica*, *G. rufobrunnea*, and *Argema mimosae*).

Amino Acid	*G. postica* (n = 6)	*G. rufobrunnea* (n = 6)	*Argema mimosa* (n = 6)
**Gly ^NP^**	21.4 ± 0.26	20.2 ± 0.13	19.8 ± 0.16
**Ala ^NP^**	9.5 ± 0.16	11.0 ± 0.10	7.2 ± 0.10
**Pro ^NP^**	1.2 ± 0.03	3.6 ± 0.02	3.3 ± 0.03
**Val ^NP^**	2.0 ± 0.04	1.9 ± 0.02	1.4 ± 0.02
**Met ^NP^**	0.5 ± 0.02	0.1 ± 0.01	0.2 ± 0.01
**Leu ^NP^**	1.1 ± 0.02	2.1 ± 0.01	1.7 ± 0.02
**Isoleu ^NP^**	1.5 ± 0.04	1.6 ± 0.01	1.3 ± 0.01
**Ser ^P^**	31.3 ± 0.17	20.4 ± 0.10	19.6 ± 0.15
**Cys ^P^**	0.3 ± 0.01	0.1 ± 0.01	0.1 ± 0.01
**Thr ^P^**	3.4 ± 0.06	3.1 ± 0.01	2.3 ± 0.03
**Asp ^aP^**	16.7 ± 0.17	13.3 ± 0.10	14.1 ± 0.16
**Glu ^bP^**	9.8 ± 0.15	9.0 ± 0.05	7.3 ± 0.07
**Arg ^P^**	4.9 ± 0.07	3.6 ± 0.02	3.4 ± 0.02
**His ^P^**	1.4 ± 0.05	1.1 ± 0.01	2.0 ± 0.01
**Lys ^P^**	1.7 ± 0.03	1.5 ± 0.01	1.5 ± 0.02
**Phe ^NP^**	2.7 ± 0.06	2.2 ± 0.05	1.8 ± 0.02
**Tyr ^P^**	3.9 ± 0.06	2.9 ± 0.06	3.0 ± 0.04
**Tryp ^P^**	-	-	-
**P:NP ratios:***G. postica* (**65:35**); *G. rufobrunnea* (**56:44**); *Argema mimosae* (**59:41**)			

**P** = polar; **NP** = non-polar; **a** = aspartic acid&asparagines; **b** = glutamic acid and glutamine.

**Table 2:** Activity (%) of the three silk sericin strains against 2,2-diphenyl-1-picrylhydrazyl radical (DPPH) and 2,2′-azinobis-(3-ethylbenzothiazoline-6-sulfonic acid) (ABTS˙^+^) free radical.

Sericin Extracts	EC_50_ Concentration [Mean ± SD] (mg/mL)	
	DPPH Radical Scavenging ABTS˙^+^ Radical Scavenging	
*G. postica*	6.39 ± 4.1	6.52 ± 2.0
*G. rufobrunnea*	5.40 ± 1.5	5.56 ± 1.3
*Argema mimosae*	2.31 ± 2.1	2.93 ± 1.8
Trolox	0.70 ± 5.3	1.09 ± 1.1

Significant difference (*p*; < 0.05).
